# Mastication of Nuts under Realistic Eating Conditions: Implications for Energy Balance

**DOI:** 10.3390/nu10060710

**Published:** 2018-06-01

**Authors:** Breanna M. McArthur, Robert V. Considine, Richard D. Mattes

**Affiliations:** 1 Department of Food Science, Purdue University, West Lafayette, IN 47906, USA; bmcarth@purdue.edu; 2 Department of Medicine, Indiana University School of Medicine, Indianapolis, IN 46202, USA; rconsidi@iupi.edu; 3 Department of Nutrition Science, Purdue University, West Lafayette, IN 47906, USA

**Keywords:** nuts, physical properties, mastication, bolus formation, swallowing, satiety

## Abstract

The low digestibility and high satiety effects of nuts have been partly attributed to mastication. This work examines chewing forces and the bolus particle size of nuts (walnuts, almonds, pistachios) varying in physical properties under different conditions (with and without water, juice, sweetened yogurt and plain yogurt) along with satiety sensations and gut hormone concentrations following walnut consumption (whole or butter). In a randomized, cross-over design with 50 adults (25 males, 25 females; Body Mass Index (BMI) 24.7 ± 3.4 kg/m^2^; age: 18–52 years old (y/o), the chewing forces and particle size distribution of chewed nuts were measured under different chewing conditions. Appetite sensations were measured at regular intervals for 3 h after nut intake, and plasma samples were collected for the measurement of glucose, insulin and Glucagon-like peptide-1 (GLP-1). The three nuts displayed different particle sizes at swallowing though no differences in chewing forces were observed. Walnuts with yogurt yielded larger particle sizes than the other treatments. Particle size was not correlated with either food palatability or flavor. Fullness sensations were higher after whole nut than nut butter consumption though there were no significant changes in glucose, insulin, or GLP-1 concentrations under any condition. Changing the conditions at swallowing might influence the release of energy from nuts.

## 1. Introduction

Nuts are high-fat, energy-dense foods that, historically, have been associated with adiposity. However, mounting evidence suggests that in the context of a healthy diet, the inclusion of nuts does not promote weight gain [1,2,3,4,5,6,7,8,9,10]. This has been attributed to their potential to increase energy expenditure [3,11,12], high satiety value [13,14,15,16,17,18,19,20], and limited energy bioaccessibility (release) [21,22,23,24]. Mastication contributes to each of these mechanisms, but in different ways. A better understanding of oral processing may therefore yield insights for manipulating nut consumption to manage energy balance.

Several studies on gum-chewing have documented that mastication elevates energy expenditure (EE) due to the muscular activity involved in chewing [25,26,27]. Chewing reportedly increases energy expenditure by 11 kcal/h [25], although more recent studies reveal a smaller increment in thermogenesis [26,28]. Other work noted a significantly larger increase in diet-induced thermogenesis (DIT) after consumption of a solid meal compared to the same meal in puree form [28]. Although mastication was not measured, multiple studies document acute effects of peanut consumption on energy expenditure [3,11,12]. Supportive findings in trials with other nuts are not robust. One study reported a 14% increment in EE after almond consumption [29], although in another report, no thermogenic response was noted [2]. Studies with walnuts [19] and hazelnuts [30] have also revealed no variation in thermogenesis. Consequently, the evidence to date is not conclusive on this mechanism. If the act of chewing does influence thermogenesis, the effect is likely small in magnitude [28].

Investigations on solid and liquid versions of high-carbohydrate, high-protein, and high-fat foods indicate that ratings of hunger and total energy intake are higher following consumption of the liquid versions of each of these foods, regardless of the energy source tested [31,32,33,34,35,36,37,38]. These findings suggest that oral processing effort/time may contribute to satiety. However, it is unclear whether this effect is direct or indirect. Some work indicates the act of chewing can enhance satiety by neural and/or endocrine mechanisms. Animal studies show that chewing directly activates satiety centers in the hypothalamus and suppresses food intake [39,40]. Additionally, oral stimulation prompts cephalic phase responses that, in turn, influence the secretion of hormones (e.g., CCK, PYY, GLP-1, insulin) that purportedly mediate appetite and metabolism [41,42]. Alternatively, food components (e.g., protein, fat, fiber) rendered bioavailable through mastication have been correlated with increases in satiety and reductions in energy intake. Recently, the satiating properties of walnuts have been ascribed to their fatty acid profile, which is especially rich in polyunsaturated fatty acids (e.g., alpha-linolenic acid) [43]. However, very few study designs isolate the independent effect of mastication on appetitive responses, so clear conclusions cannot be drawn. One aim of this study was to explore the role of mastication of walnuts on appetitive sensations and selected “satiety hormone” concentrations.

Chewing has a major role in food digestion and nutrient bioaccessibility. Chewing mechanically ruptures the cell walls of plant foods thereby freeing nutrients that may not have been accessible to the body. Randomized controlled trials exploring the relationship between mastication and energy bioaccessiblity from nuts reveal increased energy losses in the stools of subjects on diets rich in walnuts [44], pecans [21], pistachios [45], almonds [46], or peanuts [3,24]. This low bioacessibility is attributed to the resistance of nut parenchymal cell walls to degradation in the gut and inadequate mastication [47]. However, one study observed greater lipid absorption (e.g., less fecal fat excretion) after almonds were chewed 40 times versus 10 times [18], calling into question the role of increased chewing as a strategy for weight loss/maintenance. Whereas prolonged oral stimulation may enhance the signals generated for appetite control [48,49,50], the greater nutrient availability derived through chewing could increase energy absorption. Thus, from this perspective, questions remain as to whether chewing is an aid or hindrance to energy balance.

While there are similarities in nutrient composition and energy density between nuts that support viewing all types of nuts similarly, there are structural and compositional differences that challenge this view. First, nuts differ in their physical properties (e.g., hardness). Almonds for example, require a higher breaking force than peanuts [51,52]. Dissimilarities in hardness between nuts can modify masticatory behavior such as chewing duration and/or bite strength, which determine particle size, and energy bioaccessibility. Second, the context in which nuts are consumed can vary (e.g., nuts alone or in combination with foods and beverages) [53] which can impact their oral processing and availability of nutrients [54]. For example, fluid (e.g., water, clear beverages) and semi-solid (e.g., gels, yogurt) foods ingested with nuts shorten the rate/duration of chewing and trigger early swallowing of the mixture [55,56,57]. Furthermore, prandial fluid intake has been suggested to increase the palatability of a meal which can also lead to fewer chews, larger bite sizes, and an accelerated eating rate [58,59,60,61], notably when the fluid is sweetened. Thus, taking nuts with fluid products, especially sweet ones, may increase particle sizes within the ingested bolus, affecting the ultimate release of energy from the nuts. However, data concerning this topic are scarce [55,57], thus this issue was examined in this trial.

Recent evidence indicates 21% of the energy in walnuts is not bioaccessible, which is similar to the energy value for almonds (20% lower than predicted based on Atwater values or bomb calorimetry) though they have dissimilar physical properties [44,46]. Moreover, walnuts reportedly have energy yields that are lower than pistachios (5% less than predicted) with close physical properties [45]. Although the mechanisms are unclear, it may be hypothesized that nuts evoke different amounts of fragmentation and cellular disruption in the oral cavity due to differences in their physical properties [62,63], a postulate that was also examined in this study.

Overall, the goals of this study were two-fold: First, it was of particular interest to contrast the masticatory efficiency of nuts (walnuts, almonds pistachios) varying in physical properties as this may result in changes in pre-swallowing particle size and, consequently, their digestion. Additionally, we investigated the influence of adding high water foods and beverages of varied flavors (sweet vs. plain) to walnuts on masticatory performance and pre-swallowing particle size. We hypothesized that coupling walnuts with a sweet flavor would increase palatability and reduce masticatory efficiency, resulting in larger particle sizes in the swallowed bolus. Additionally, mixing walnuts with fluid foods (liquids and semi-solids) was expected to facilitate swallowing of larger particles. Secondly, we investigated the effects of walnut consumption as whole nuts or butter on appetitive sensations and gut hormone secretion. We hypothesized that the whole nuts would elicit a higher satiety value compared to the nut butter. Differences from other tree nuts in chemical (e.g., fatty acid and antioxidant profile) and physical characteristics make walnuts an intriguing target for the study of the contribution of oral processing to their satiety and low energy yield. Moreover, given that walnuts uniquely provide nutrients associated with various health benefits [64], evaluating how these nuts are orally processed alone and under realistic eating conditions is worthwhile, especially since their health impact may be altered by the efficiency of mastication.

## 2. Materials and Methods

### 2.1. Participants

Fifty healthy adults (25 M, 25 F; BMI 24.7 ± 3.4 kg/m^2^ (range: 19.7–33.7 kg/m^2^); 25 ± 8 y/o (range: 18–52 years) were recruited through public advertisements. Eligibility criteria included healthy dentition and no nut allergies. All participants were non-smokers and were not taking medications known to affect the study outcomes. Each participant signed an informed consent form approved by the Purdue University Institutional Review Board and received monetary compensation for participation.

### 2.2. Experimental Design

**Figure 1:**
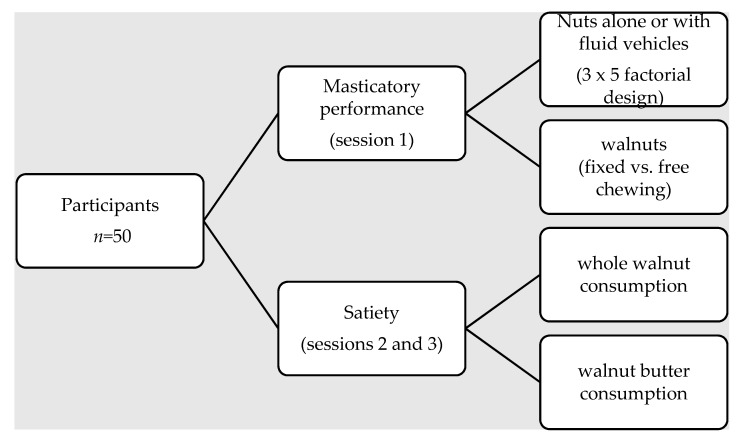
Study design flow diagram.

The study followed a within-subject experimental design. Two experiments were carried out in three separate testing sessions. In experiment 1, each participant participated in one session of masticatory performance (Figure 1). Participants reported to the laboratory after having refrained from eating and using oral care products for at least 2 h. Participants were presented 5 g portions of nuts (walnuts, almonds, pistachios) with and without water, apple juice, plain yogurt or sweet yogurt in a randomized order. They were instructed to chew each sample, one at a time, at a constant rate (timed to a metronome at a rate of 1 chew/s for 15 s. Each sample was then expectorated into a pre-weighed plastic container and rated for palatability on a scale of 1 to 9 with 1 = “dislike extremely” and 9 = “like extremely [65].” A separate chewing condition was applied where participants were instructed to chew the walnuts for a predetermined number of chewing cycles (15 s) or until ready to swallow followed by expectoration into pre-weighed containers. For all treatments, electromyography (EMG) activity was recorded throughout the chewing sequences and particle size was determined by wet sieving.

Experiment 2 was divided into two sessions separated by approximately 1 week (Figure 1). Participants reported to the laboratory in the morning after an 8 h fast. They rated their appetitive sensations upon arrival using a visual analogue scale (VAS) presented on a personal digital tablet. Standard appetite questions were used as described previously by Hill et al [66]. After completing the VAS, a catheter was inserted in a vein in the antecubital space of the arm. Following a 10 min acclimatization period, a second appetite questionnaire was completed and a baseline blood sample (time point = 0) was drawn. Participants were then presented with 28 g of whole walnuts (raw) or walnut butter (whole raw walnuts ground to smooth butter consistency by a standard food processor) in a counterbalanced order. Immediately following walnut consumption, blood samples were drawn at 15, 30, 45, 60, 120 and 180 min. Blood samples were collected in EDTA-coated tubes containing DPP-IV inhibitor on ice, and centrifuged to separate the plasma. Plasma was aliquoted and initially frozen at −20 °C prior to storage at −80 °C Plasma GLP-1active was measured in duplicate using a commercially available ELISA kit (Millipore). All samples for an individual were run on the same ELISA plate. Glucose and insulin concentrations were determined using a Roche Cobas Integra Analyzer. 

### 2.3. Test Foods

Three whole nuts were evaluated: Walnuts (raw unsalted, Sacramento, CA, USA), almonds (roasted salted, Sacramento, CA, USA), and pistachios (dry roasted, Kraft Heinz Foods Company, Chicago, IL, USA). Each type of nut was drawn from a single batch and was stored in sealed containers in a refrigerator at 4 °C until the day of testing. Each nut sample weighed ~5.0 g. Five eating conditions were assessed: Nuts alone, nuts with water (deionized), nuts with apple juice (Mott’s 100% Apple Juice, Mott’s LLP, Plano, TX, USA), nuts with sweet yogurt (Greek Gods Greek Yogurt Honey Vanilla, Hain Celestial Group, Inc. Lake Success, NY, USA) and nuts with plain yogurt (Greek Gods Greek Yogurt Traditional Plain, Hain Celestial Group, Inc., Lake Success, NY, USA).

### 2.4. Breaking Force

Texture analyses for each of the three whole nuts was conducted using a TA XT2 Texture Analyzer (Stable Micro Systems, Godalming, UK) fitted with a knife probe and set to penetrate the samples to a depth of 4 mm (almonds, pistachios) and 8 mm (walnuts) at a speed of 1 mm/s. Two different penetration depths were applied since samples differed in shape and dimension (e.g., thickness). Twenty replicates were performed for each nut type and a mean value was calculated.

### 2.5. Masticatory Performance

The microstructure of chewing was characterized by electromyographic (EMG) recording (BioPac Systems, Inc., Goleta, CA, USA). The temporalis and masseter muscles on the dominant chewing side of each participant were identified by palpation and bipolar surface electrodes were placed approximately 3 cm apart along each muscle. A ground electrode was placed on the inside of the participants’ opposite wrist. Four parameters were quantified: Maximum bite force (volts); mean bite force (volts); total muscle work (area of the EMG signal); and total number of chews. The raw EMG output was rectified due to the bipolar nature of the signal.

### 2.6. Proportional Particle Size Distribution

A total of 800 boluses were collected (50 subjects × 16 samples) and particle size was determined by sieving the expectorated boluses through a stack of pre-weighted sieves. The mesh sizes were: >3.35 mm, 3.35–2.0 mm, 2.0–1.0 mm, 1–0.50 mm, 0.50–0.25 mm, 0.25–0.125 mm and <0.125 mm (WS Tyler, Mentor, OH, USA). The sieves were arranged in descending order of mesh size. Because wet bolus particles tended to stick together, a 0.1% sodium chloride solution was poured over the expectorated samples and allowed to drain completely through the stack of sieves. The sieves and expectorated samples were then dried for 17 h at 74 °C in an air-dry oven to eliminate all the water. This time/temperature was selected using previously described methods [55,67], with the noted modifications.

### 2.7. Statistical Analysis

Statistical analyses were performed using SPSS version 22.0 (IBM Corp., Armonk, NY) The Kolmogorov-Smirnov test was used to verify data distribution normality. Non-parametric tests were used when the assumptions of parametric tests were not met. A non-parametric Wilcoxon signed-rank test was applied to compare the parameters describing mastication. Repeated measures analysis of variance (ANOVA), with nut type and condition as within subject factors, were applied to test if significant differences exist between nuts and between conditions on palatability ratings, masticatory performance, and proportional particle sizes. Repeated measures mixed models were used to test the overall condition effect (i.e., liquids vs. solid vs. semi-solids, sweet vs. plain) on the particle size distribution. When significant interactions were observed, main effects were tested using paired *t*-tests. Spearman’s correlation analysis was performed to examine the relationship between masticatory performance, particle size, and palatability. Repeated measures ANOVA and mixed models were also used to test the effects of nut form (whole vs. nut butter) on appetite and post-prandial responses. When appropriate, *post hoc* comparisons were made with Bonferroni adjustments. Statistical tests were performed at a significance level of *p* < 0.05.

## 3. Results

### 3.1. Nut Breaking Force

The instrumental breaking force was the lowest for walnuts (1088 ± 177 g), followed by pistachios (1833 ± 169 g) and almonds (3395 ± 149 g).

### 3.2. Mastication Parameters

**Figure 2:**
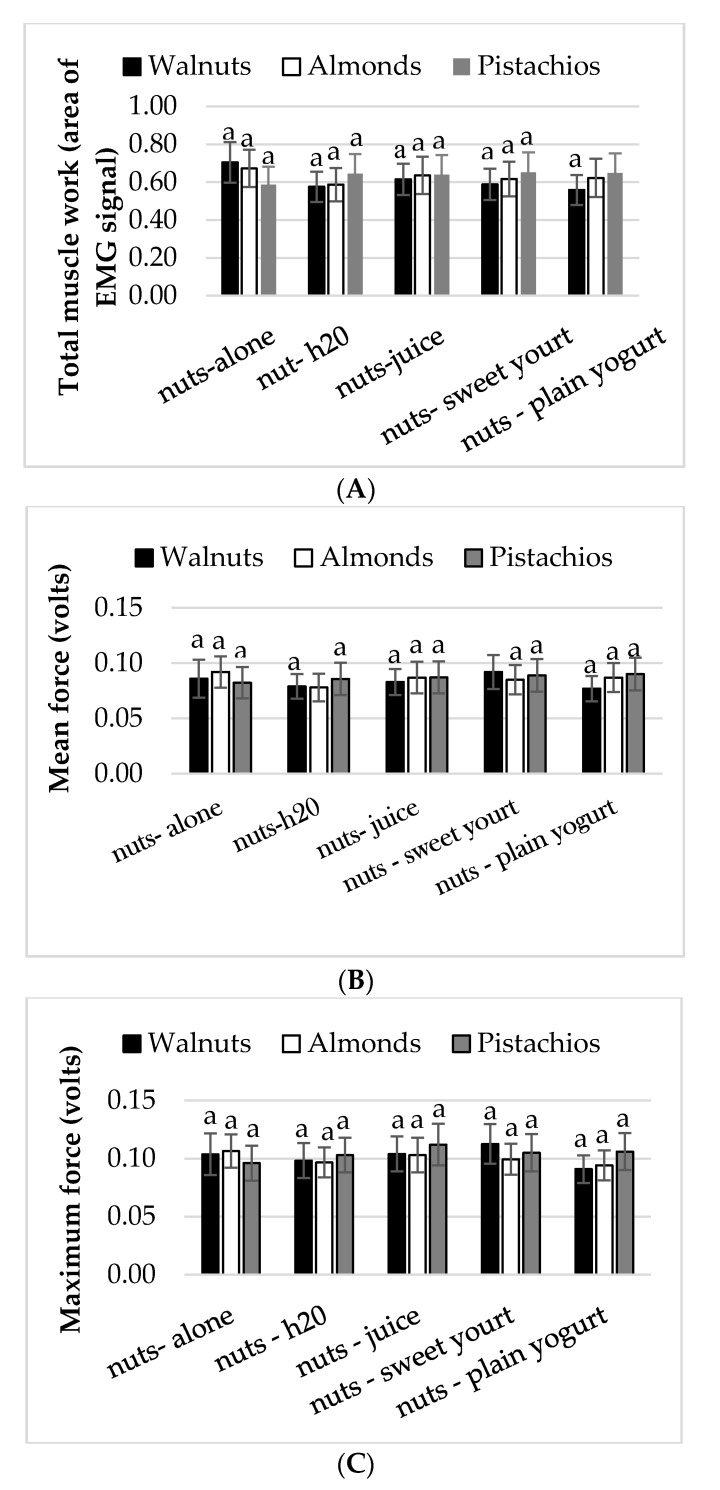
Mean ± S.E.M. (**A**) total muscle work (AREA), (**B**) mean bite force, and (**C**) maximum bite force obtained from EMG recordings. Conditions with the same lower case letters (**a**) represent no significant differences between conditions (*p* > 0.05).

There were no significant differences in chewing outcomes: Mean force, maximum force, and total muscle work (AREA) between eating conditions and nut types (Figure 2A–C). Similarly, for walnuts in the free and fixed chewing conditions, the total muscle work (*z* = −1.371, *p* = 0.170), mean (*z* = −0.475, *p* = 0.635) and maximum bite force (*z* = −0.005, *p* = 0.996) were comparable (data not shown). The Wilcoxon signed-rank test revealed that chewing time (second) increased when walnuts were chewed and expectorated at the time participants felt the need to swallow (20 s) compared to when walnuts were chewed for a fixed time (15 s), (*z* = −4.583, *p* < 0.0005).

### 3.3. Particle Size

**Figure 3:**
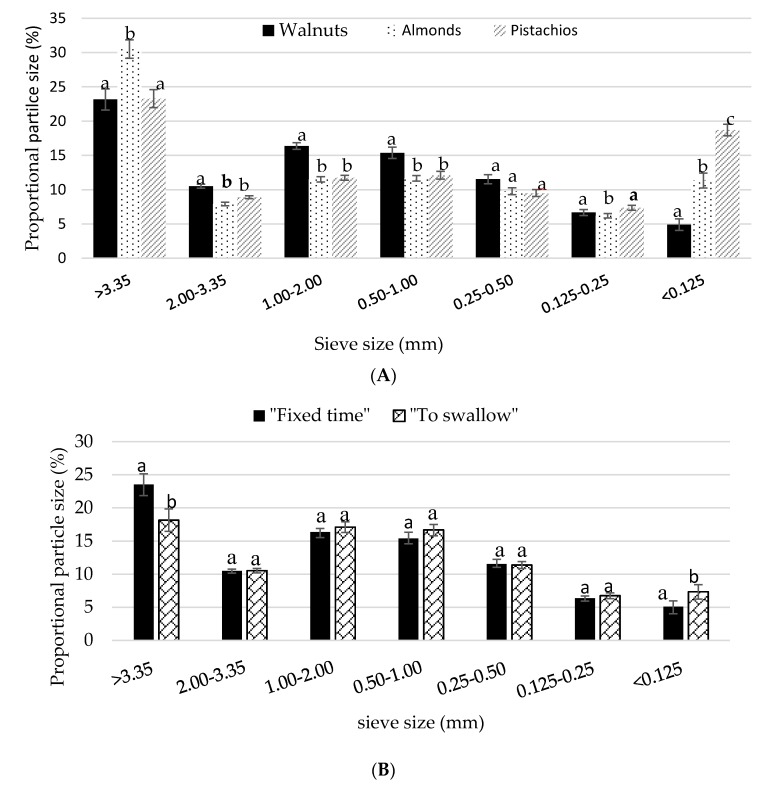
(**A**) Mean ± S.E.M particle size distribution of nuts chewed in isolation. Comparisons are based on two-way repeated measures ANOVA with *post hoc* Bonferroni multiple comparison test. Different lower case letters denote significant differences between nuts (*p* < 0.05); (**B**) Mean ± S.E.M particle size distribution by size of walnuts chewed for a fixed time and to the point of swallowing. Letters that are different denote significant differences between mastication protocols (*p* < 0.05).

There was a moderate, but significant effect of nut (*F* (2, 80) = 16.096, *p* < 0.0001) and condition (*F* (3, 139) = 6.906, *p* < 0.0001) on the recovered food mass collected after mastication. The percent recovery was slightly, but significantly lower for the walnuts (88.4 ± 1.1%) compared to the almonds (91.0 ± 0.94%) and pistachios (92.8 ± 0.87%) *(p* < 0.01). The proportion of particles larger than 3.35 mm were significantly greater in almond boluses compared with walnuts and pistachio boluses (*p* < 0.01). The share of particles larger than 3.35 mm was comparable between the pistachios and the walnuts (*p* > 0.01). In contrast, pistachios had a higher proportion of particles less than 0.125 mm compared with the other nuts (*p* < 0.01) (Figure 3A). The proportion of particles less than 0.125 mm was significantly larger for walnuts chewed until the point of swallowing than the walnuts chewed for a fixed time (*p* = 0.003) (Figure 3B).

In an analysis that examined the main effect of condition type (e.g., nut alone, water, juice, sweet yogurt, plain yogurt) at each sieve size level, particles were larger (>3.35 mm) with sweet and plain yogurt compared with the nut alone (*p* < 0.01).

The ANOVA revealed a main effect of condition on particle size (*F* (3140) = 8.358, *p* < 0.0001). A larger proportion of particles less than 0.125 mm were detected in boluses with water than corresponding boluses without water (*p* < 0.01). Boluses with juice contained more particles less than 0.125 mm than boluses with plain yogurt (*p* < 0.01). In a second analysis that examined the overall condition effect (e.g., solid nuts alone vs. with liquid beverages vs. with semi-solid yogurts) on particle size, a high proportion of bolus particles greater than 3.35 mm was found with the yogurts and the beverages than with the nuts alone (*p* < 0.01).

**Figure 4:**
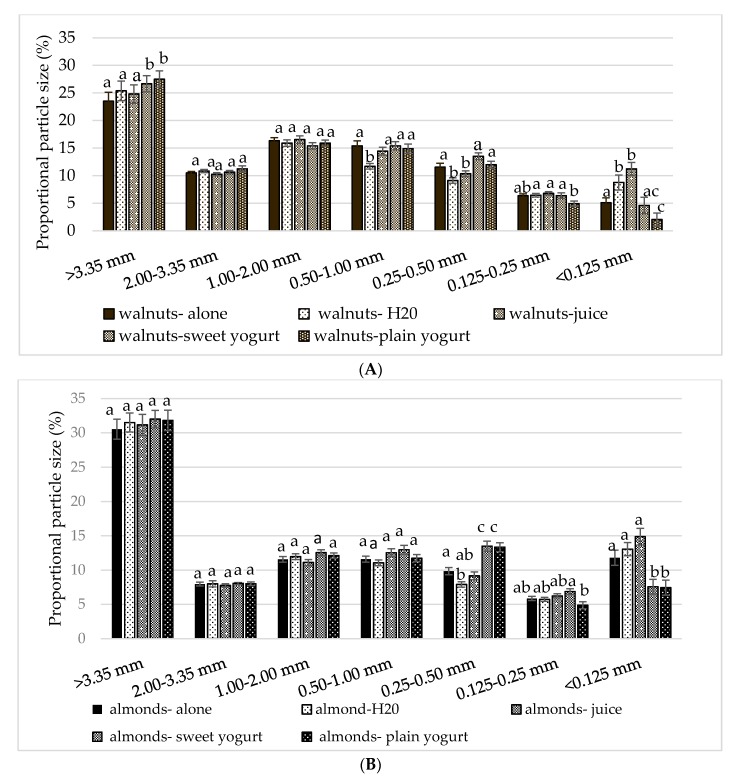

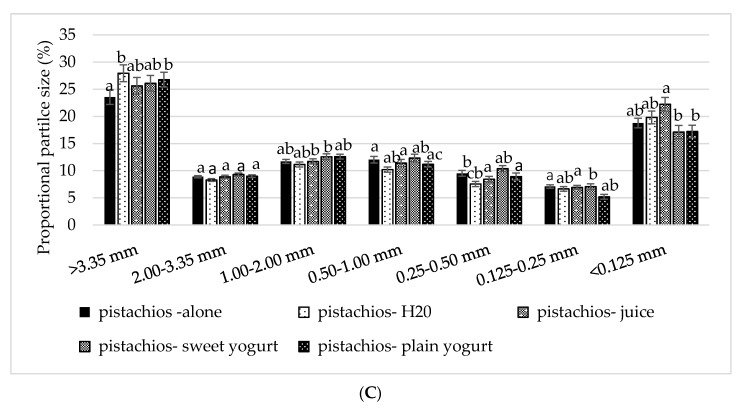
Mean ± S.E.M particle size distribution by condition for nuts: (**A**) walnuts, (**B**) almonds, and (**C**) pistachios. Comparisons are based on two-way repeated measures ANOVA with *post hoc* Bonferroni multiple comparison test. Different lower case letters denote significant differences between conditions (*p* < 0.05).

Significant condition-by-sieve (*F* (10, 444) = 18.039, *p* < 0.0001) and nut-by-sieve (*F* (4202) = 104, *p* < 0.0001) interactions were noted (*p* < 0.01). *Post hoc* analyses indicated that walnut particles were significantly larger (>3.35 mm) with the sweet and plain yogurt compared to the walnuts alone (*p* = 0.005 and *p* = 0.002, respectively) (Figure 4A). Similarly, there was a significantly greater proportion of pistachio particles >3.35 mm with plain yogurt and water compared to alone (*p* = 0.009 and *p* < 0.001, respectively) (Figure 3B). Significantly more pistachio particles were <0.125 mm with juice compared with sweet or plain yogurt (both *p* < 0.01). Almonds with both sweet and plain yogurt resulted in a higher proportion of particles 0.25–0.50 mm compared to all other almond treatments (*p* < 0.01) (Figure 4C).

Across all nuts, overall sweetness had no effect on large particle sizes (*p* > 0.01). There was a significant effect of sweet flavor on particles less than 0.50 mm (*p* < 0.01). The proportions of particles in the three smallest sieves were higher in the sweet compared to the plain conditions (*p* < 0.001). This effect was independent of the fluid form (e.g., liquid beverage or semisolid yogurt).

### 3.4. Palatability Ratings

Mean preference scores were 6 ± 0.31, 6 ± 0.30, 6 ± 0.44, 7 ± 0.31, 5 ± 0.39 for nuts alone, nuts with water, nuts with juice, nuts with sweet yogurt, and nut with plain yogurt, respectively. There was no main effect of nut type on preference scores, although there was a significant condition effect (*F* (3, 149) = 13.5, *p* < 0.0005). Nuts ingested with sweet yogurt were rated as more palatable than nuts with plain yogurt, water, and juice (*p* < 0.0005). Palatability ratings did not correlate with masticatory performance or particle size.

### 3.5. Effect of Walnut Consumption on Metabolic Measure

**Figure 5:**
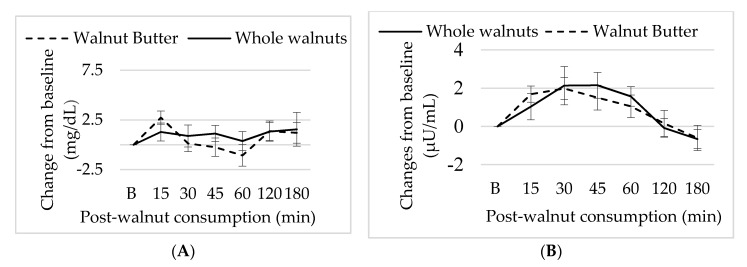
Mean ± S.E.M changes in (**A**) insulin and (**B**) glucose concentration subsequent walnut consumption.

Baseline glucose and insulin concentrations were not different between sessions. There were no significant effects of nut form or time on plasma glucose or insulin concentrations following ingestion (*p* > 0.05) (Figure 5).

### 3.6. Appetitive Sensations

**Figure 6:**
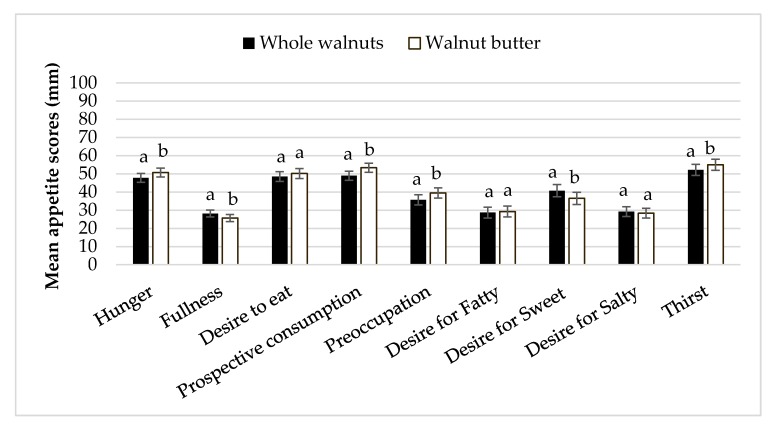
Mean ± S.E.M appetite indices subsequent walnut consumption. Letters that are different denote significant differences between nut form (*p* < 0.05).

Appetitive sensations at baseline did not differ between testing sessions. There were significant differences in fullness and hunger ratings between time points and treatment groups. Mean fullness was higher and hunger was lower with the whole walnut treatment compared to walnut butter (*p* < 0.05). Ratings of fullness were higher than baseline after 15 and 30 min (*p* = 0.014 and *p* = 0.019, respectfully) and hunger ratings were suppressed below baseline 15 min after whole walnut intake (*p* = 0.011). Additionally, preoccupation with food and thirst was significantly lower with the whole walnuts than with the walnut butter (*p* = 0.006). Desire for something sweet was lower with the walnut butter than the whole walnuts (*p* < 0.0005) (Figure 6).

### 3.7. Gut Hormones

**Figure 7:**
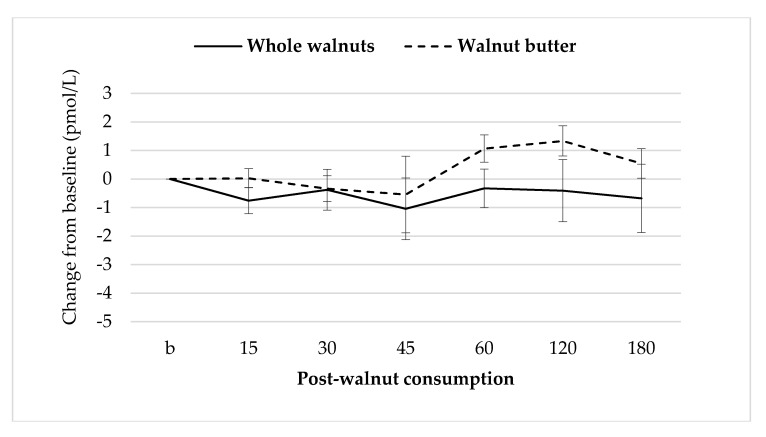
Mean ± S.E.M changes in GLP-1 concentrations after walnut consumption.

Baseline GLP-1 concentrations were not different between sessions. There were no significant treatment effects on GLP-1 concentrations (Figure 7).

## 4. Discussion

The primary purpose of mastication is to ensure solid food particles are reduced to a size that can be effectively incorporated into a bolus and safely swallowed. Secondarily, there is evidence that particle size is a determinant of energy and nutrient bioacessibility [68,69]. The optimal size is a function of an individual’s anatomy, the nature of the food, and the conditions under which it is ingested [70,71]. Though nuts are viewed as a single class of foods with physical properties that are more similar than dissimilar, the clinical evidence suggests they are not processed equivalently under a given set of conditions or across varying conditions. This is substantiated by the present findings. 

The first part of this work aimed to study the efficiency of mastication during the chewing of different types of nuts. Variations in muscle activity have been reported for different types of nuts [51,52], and other foods such as meat [72], and rice [73]. Those observations generally indicate that harder samples require greater initial and mean bite forces. Additionally, hard foods tend to elicit a higher number of chewing cycles relative to soft foods [70,74]. Mastication indices were measured here to explain possible contributions to changes in particle size across three types of nuts. Based on physical properties determined instrumentally, we predicted a rank ordering of almonds > pistachios > walnuts (largest to smallest particle size). However, in the fixed chewing condition, the observed ordering was almonds > walnuts = pistachios. Our observation is not necessarily an outlier as other work has revealed an inverse association between food hardness and mean bolus particle size [75]. Additional food properties, such as food/particle shape (e.g., elongated vs. spherical particles), structure, cohesiveness, and elasticity also have an influence on mastication [76]. It is likely these additional properties attenuated the effects of nut hardness on particle size. Thus, in this study, measured masticatory intensity (initial and mean bite force) was not predictably related to the hardness of the nuts or ultimate particle size.

This study also assessed whether dietary context influences the oral processing of nuts, since nuts are commonly consumed in conjunction with other foods and beverages. A liquid and semi-solid were chosen as test vehicles mainly because they have been shown to decrease chewing and accelerate swallowing [56,57,77,78,79]. For this reason, it was anticipated that adding a fluid or semi-solid to nuts would result in reduced muscle activity, leading to reduced chewing efficiency and larger particle sizes at swallowing. However, there were no differences in measured chewing indices between any of the conditions tested. This finding differs from some earlier studies [55,80,81], but it agrees with studies by Derks et al. (2015), who did not find differences in chewing behavior between different types of liquid and semi-solid stimuli [82]. Other studies have shown that the addition of fluids lowers the chewing forces applied to solid foods, however the decrease in muscle activity was accentuated for soft solids (cakes and toast) rather than hard solids (peanuts) [81]. With hard solids, the fluid stimuli had a larger influence on the number of chewing cycles than on muscle activity [81]. Additionally, the findings might be explained by the different ratios of nut to semi-solid/liquid used in the different studies. In a chewing study where brazil nuts were suspended in yogurt in varying concentrations, both the number of chews made before swallowing and the time needed to swallow increased significantly with concentration [57].

In terms of particle size, analyses showed significant effects of the addition of liquid and semi-solid vehicles. This is consistent with a number of previous studies [55,80,81]. With liquid water, bolus particles were smallest (<0.125 mm) compared to all other conditions, possibly due to its low viscosity, which increases sensations of roughness by moistening and separating particles [83]. Another possible explanation for this may be that chewing in the presence of water elevates suprahyoid muscle activity, which coordinates tongue movements, including compression between the tongue-palate [82]. Boluses contained a higher proportion of large particles (>3.35 mm) with the semi-solid yogurts compared to masticating the nuts alone. The lubricating effects of yogurt may have masked the perceived size and roughness of the larger particles thereby lowering masticatory efficiency [84]. Additionally, embedded in a semi-solid matrix, soft- and round-shaped particles are perceived to be smaller in the mouth than harder particles of the same size range [85], possibly resulting in less chewing. As expected, the walnuts with yogurt gave rise to larger particle sizes than chewing the nuts separately. Similarly, pistachios with yogurt resulted in large particle size, whereas the almonds with yogurt yielded particles of smaller sizes. These results could be explained by the oral viscosity and flow properties of yogurt [86], which may preferentially select larger particles for chewing (oral selection) during the early stage of mastication, thus increasing their chance for fragmentation and hindering their swallowing [87]. Additionally, the lubricating properties of a semi-solids reportedly deteriorate with inclusion of hard particles, leading to increased mastication. This could explain the smaller observed particle sizes in the almond-yogurt mixture. Therefore, the present study confirms that properties of liquids and semi-solids influence swallowing decisions between nuts.

Sweet flavor is an important oral sensory property proposed to decrease oral residence time, and accelerate swallowing [60,88]. Therefore, the sweet foods were expected to decrease the chewing efficiency of walnuts, resulting in larger particle sizes in the swallowed bolus. While no differences were observed on the proportion of large particle sizes between the sweet and plain complementary foods, there was a greater proportion of small particles (less than 1 mm) with sweet vehicles than plain vehicles. Because oral movements increase in response to a sweet flavor [89], this may have led to an improved efficiency in the breakdown of the particles. This finding does not support our initial hypothesis on sweet flavor and it is different from previous studies [59,90]. There are multiple possible explanations for this. First, part of the effect noted here may stem from the fact that the sweet stimuli were fluids and semi-solids and the latter property may have dominated. Fluids increase the intensity of shear/squeezing between oral contact surfaces, (e.g., teeth-teeth, teeth-tongue, and tongue-palate) leading to more food fracturing [91]. Second, the sweet as well as acid content (citric acid) in juice may have enhanced salivary flow rates, more than yogurt, which may have resulted in an improved masticatory efficiency (e.g., smaller particle sizes compared with yogurt) [92]. Thus, the present findings provide suggestive evidence that sweetness results in a bolus with smaller, rather than larger particles.

Previously it was reported that changing the palatability of a meal has marked effects on masticatory function (e.g., chewing rhythm, eating rate, overall intake) [58,59,93], therefore we considered the effect of palatability on the mastication of walnuts. The prediction was that enhancing the palatability of nuts would result in a reduction in chewing behavior, leading to larger particle sizes at swallowing. However, we did not observe an independent effect of palatability on chewing indices or particle size, possibly because the observed differences in palatability between conditions was limited and all were rated positively. Similar findings have been reported in studies with peanuts [52] and almonds [51]. Earlier studies on the microstructure of eating, documented an effect of palatability on masticatory behavior [58,59,93], but the effects appeared to be food-specific and the stages/duration of mastication were not examined. So the independent effect of palatability on oral processing remains uncertain.

The second part of this work was aimed at studying the role of mastication on the high satiety capacity of walnuts. Regular intake of walnuts generally [19], but not uniformly [94,95], promotes strong fullness sensations. The reason for the discrepancy in published findings is not apparent, but may relate to methodological variations. For example, the study reporting high fullness assessed sensations after three days of chopped walnut intake relative to no nut intake in a controlled environment with participants that had metabolic syndrome. Whereas the studies failing to observe strong fullness ratings assessed satiety in people with overweight and obesity following a reduced-energy diet with whole walnuts and a reduced-energy diet without walnuts over 6 months in a free-living environment. In the present study, intake of whole walnuts elicited greater fullness compared to the butter form among individuals who are lean. This would suggest that chewing has an influence on appetite, possibly dependent on weight status [96].

The change in appetite with walnut consumption could not be ascribed to the release of GLP-1 or insulin, two reported satiety hormones. No correlation was observed between masticatory indices and concentrations of these hormones in the present study. The lack of effect of endocrine signals on appetite has been reported previously in other nut studies [19,43,97]. Walnuts and other nuts, generally, have not been effective stimuli for gut peptide secretion [16,17,18,19,95], but impart strong satiation/satiety effects. The present study supports the hypothesis that the observed differences in appetite may be attributable to mastication, which may exert its effect directly through neural rather than endocrine mechanisms [98]. 

A strength of the current study is the cross-over design. Other work has documented marked inter-individual variability in mastication and this hampers identification of treatment effects. Another strength is that the results facilitate understanding of the oral processing of walnuts and other nuts under ecologically valid conditions. In line with previous results [55], we found that changing the eating conditions of nuts, including walnuts, affected pre-swallowing particle size. One limitation of this trial is that we standardized the number of chews for each treatment during the assessment of masticatory performance mainly because of the subject-to-subject variation in habitual chewing rates and times [99]. By controlling the chew time/rate the bolus may not faithfully reflect its state at the point of swallowing under natural conditions. Evaluating the oral processing of all treatments under a fixed and experimental controlled condition would have been useful. Another limitation is that we only measured short-term satiety, which may not be predictive of long-term ingestive behavior. A previous study observed increased fullness following a walnut breakfast only after the third and fourth day of the intervention [19]. The authors concluded that the mechanisms responsible for the satiety effects of walnuts may not manifest over a short time period.

## 5. Conclusions

In summary, the breakdown pattern for nuts differed and was not explained by a sole physical property (hardness). Breakdown is likely determined by multiple food properties (elasticity, plasticity, shape, etc.), consumer variations (e.g., dentition, swallowing threshold) and the conditions under which the nuts are consumed (e.g., complementary food viscosity, taste quality). In contrast to some prior findings with other foods [61], sweet flavor was associated with a greater preponderance of small particle sizes. Further study will be required to determine if this is a specific effect with nuts.

Fullness increased after the mastication of whole walnuts compared to walnut butter, though gut peptide concentrations remained unchanged. The present findings raise the question of whether the differences in oral processing translate into altered digestive processes [100]. Additional studies are warranted to fully understand the significance of these results on both the bioaccessibility and bioavailability of energy from walnuts.
